# An Analysis of Factors Associated With Older Workers’ Employment Participation and Preferences in Australia

**DOI:** 10.3389/fpsyg.2018.02524

**Published:** 2018-12-19

**Authors:** Jack Noone, Angela Knox, Kate O’Loughlin, Maria McNamara, Philip Bohle, Martin Mackey

**Affiliations:** ^1^Centre for Social Impact, UNSW Business School, University of New South Wales, Kensington, NSW, Australia; ^2^The University of Sydney Business School, The University of Sydney, Sydney, NSW, Australia; ^3^Ageing, Work and Health Research Unit, Faculty of Health Sciences, The University of Sydney, Sydney, NSW, Australia; ^4^Cerebral Palsy Alliance Research Institute, Discipline of Child and Adolescent Health, Children’s Hospital at Westmead Clinical School, Faculty of Medicine and Health, The University of Sydney, Sydney, NSW, Australia; ^5^Tasmanian School of Business and Economics, The University of Tasmania, Hobart, TAS, Australia

**Keywords:** older workers, employment participation, work-life conflict, work centrality, person-job fit, early retirement factors

## Abstract

Australian government and organizational age-management policies continue to target employment participation among older workers in light of an aging population. Typically, efforts to reduce early retirement among older workers have focused on well-established factors, including the promotion of worker health, reducing injury, supporting caregivers, reducing age discrimination and enhancing skill development. This research extends on the former approach by examining established factors along with important emerging factors, namely work-life conflict, work centrality and person-job fit. Additionally, the research analyses the effects of gender and financial pressure on older workers’ employment participation and preferences. Logistic regression analysis of cross-sectional survey data involving 1,504 Australians aged 45–65, revealed that two established factors, physical health and caregiving, and all three emerging factors were associated with employment participation and preferences to be employed. However, important variations on the basis of gender and financial pressure were also identified. Caregiving was more strongly associated with the preference to remain employed for men (OR = 0.2.54, *p* < 0.01) than women (OR = 1.03, *ns*) and person-job fit was more strongly associated with the preference to remain employed for women (OR = 1.64, *p* < 0.001) than men (OR = 0.91, ns). Work-life conflict was more strongly associated with the preference to leave employment for those reporting limited financial pressure (OR = 0.60, *p* < 0.001) compared to those in poorer financial circumstances (OR = 0.87, *ns*). These findings suggest that organizational age management policies should focus on both established and emerging factors, particularly the provision of flexible working conditions and improving the psychosocial work environment. However, such efforts should carefully consider the different needs of men and women, and those under varying levels of financial stress. With respect to government policy to promote employment participation, the findings support a stronger focus on improving physical and psychosocial work conditions rather than increasing the pension eligibility age. This may require further collaboration between government and employers.

## Introduction

The predicted economic costs of population aging have stimulated the development of government policies to prolong employment in developed countries (e.g., Australian Government, 2011; Kryńska et al., 2014). For organizations, this has led to the development of age-management policies and practices to address well-established barriers to employment for older workers, including poor health, workplace injury, age discrimination, caregiving responsibilities and skill deficits. However, organizations have paid less attention to a range of emerging factors, namely work-life conflict, work centrality and the “fit” between a worker and their job, which may prove important to promoting workforce participation. Additionally, prior research has taken insufficient account of how these factors may differentially affect men and women or people experiencing different levels of financial pressure. This research addresses these increasingly important issues by examining how established and emerging factors contribute to older workers’ employment participation and preferences and the extent these relationships are moderated by gender and financial pressure.

In examining the role of financial pressure on older workers’ employment participation and preferences, we draw on data that was collected immediately following the 2007–2008 Global Financial Crisis (GFC). The economic constraints imposed by the GFC restricted older workers’ choices to retire or remain employed. This constraint was compounded by subsequent increases in the eligibility age for Australia’s means-tested age pension. Although Australia fared better than many countries in the GFC due to strong financial regulation and stimulus packages (Parliament of Australia, 2009), there was still a 26% drop in superannuation holdings by 2009, making retirement unaffordable for many (Kendig et al., 2013). Consequently, many older workers considered staying in the workforce longer or returning to work if already retired (Humpel et al., 2010; O’Loughlin et al., 2010; Higgens and Roberts, 2011). A recent report on employment patterns in OECD countries also notes a reversal in a previous trend toward early retirement, with a rise in the employment rate for the 55–64 age group on average, from 48% in 1990 to over 59% in 2016 (Martin, 2018).

As Australia recovered from the GFC, the pension age was set to progressively increase from 65 to 67 and potentially to 70 (Hockey, 2015). However, the average intended retirement age for current workers is 65, with only 20% expecting to work to 70 or beyond (Australian Bureau of Statistics, 2017). These figures suggest a period of approximately 5 years during which “early” retirees will be reliant on sickness or caregiving benefits (approximately two-thirds of pension payments), or personal finances, while waiting for pension eligibility. It is, therefore, important to reduce the barriers to employment participation through organizational age-management policies as well as government legislation and policies (e.g., Australian Government, 2016 – The Carer Recognition Act 2010). As noted in a recent review by Carlstedt et al. (2018), research on organizational-level factors associated with employment participation after pensionable age is scattered, and information on societal level predictors is scarce. This study aims to address this gap.

This study contributes new evidence regarding the significance of established and emerging factors on older workers’ employment participation and preferences, according to gender and financial pressure, with the findings having significant policy implications for organizations and governments. These established and emerging factors fit within four broad categories identified in recent theorizing and meta-analysis (Fisher et al., 2016; Topa et al., 2018): Family pull, work-related, person-job related and individual factors, as discussed in the following section. Topa et al.’s (2018) meta-analysis showed that each factor had a similar (bivariate) association with early retirement; the current research builds on this analysis by examining these dimensions in a multivariate context.

### Established Factors and Workforce Participation

A well-established body of literature has identified poor physical and mental health (individual factors), injury, age discrimination (work-related factors) caregiving (Family pull) and skill and training deficits (person-job related) act as barriers to employment participation for older workers. We refer to (and examine) these as established factors not only because of their evidence base, which is discussed below, but because they are formally targeted by Australian Government and organizational policies (e.g., the Australian Government, 2018 – Age Discrimination Act 2004).

#### Health

Meta-analysis of longitudinal data (van Rijn et al., 2014; Fisher et al., 2016) indicates that poor physical health is consistently associated with unemployment, transition to disability pension and early retirement. Similar outcomes are also found for poor mental health (e.g., Laaksonen et al., 2012; Paradise et al., 2012; van Rijn et al., 2014). Some scholars also estimate that rates of early retirement due to poor health will climb due to projected increases in chronic disease (Schofield et al., 2015).

#### Injury

Workplace injuries, particularly musculoskeletal disorders, are associated with disability and unemployment (Johnston et al., 2015), particularly as the severity of the injury increases (Kuhlman et al., 2014). In Australia, approximately 12% of women and 21% of men over 45 retire due to injury, disability or sickness (Australian Bureau of Statistics, 2017). Australian workers between 50 and 65 have the highest incidence of serious injury compensation claims (approximately 10.75 claims per 1,000 employees) compared to other age groups, and the prevalence is increasing over time (Safe Work Australia, 2016). Systematic reviews also demonstrate that the likelihood of returning to work following an injury decreases significantly with age (Cancelliere et al., 2016).

#### Age Discrimination

Workplace age discrimination “can occur in the form of biased decision making, negative evaluations, and unfair behaviors in contexts such as recruitment, personnel selection, performance appraisal, promotion decisions, and training” (Zacher and Steinvik, 2015, p. 327; Australian Government, 2018). Age discrimination is a significant barrier to employment (O’Loughlin and Kendig, 2017; O’Loughlin et al., 2017). Perceived age discrimination at work is also associated with long-term sickness absence (Viitasalo and Nätti, 2015), lower work engagement (Bayl-Smith and Griffin, 2014) and earlier intended retirement age (Zaniboni, 2015).

#### Caregiving

Evidence shows that caregivers work fewer paid hours, retire earlier and have poorer health than non-carers (Australian Human Rights Commission, 2013; O’Loughlin et al., 2017). A longitudinal study of 8,000 older British workers showed that, for females, entering a caregiving role increased the odds of exiting the workforce 2.64 times for part-time workers and 4.46 times for full-time workers (Carr et al., 2016). Moreover, caregiving responsibilities are also expected to intensify with population aging, leading to even earlier retirements (Yeandle and Buckner, 2017).

#### Job Skills and Training

Reduced training and opportunities for skill development have been associated with premature workforce exit (Temple et al., 2012; Barslund, 2015). One German longitudinal study showed that training programs were especially effective in prolonging employment for women on lower wages as they helped expand their earning potential (Berg et al., 2017). Another longitudinal study in Germany indicated that training for older workers was increasing, and that there was a positive association between training and job satisfaction (Zboralski-Avidan, 2015); which in turn is an important determinant of longer working life (Mein et al., 2000). According to a recent study of 3,000 Australians, older workers have an unmet need for training (Adair et al., 2016). Approximately one-third of those surveyed reported being unable to undertake further training due to employer reluctance, affordability and competing work commitments.

### Emerging Factors and Workforce Participation

In addition to the established factors discussed, several additional, emerging factors may be important to extending working life, namely person-job fit, work-life conflict and work centrality. We selected person-job fit because it directly assesses the person-job dimension outlined by Topa et al. (2018). Likewise work-life conflict is a direct indicator of family pull and can be directly influenced by organizational and government policy. Finally, we examined work centrality because it cuts across all four dimensions. It is an individual or attitudinal factor which, if high, implies that other dimensions of life (e.g., family) are less central. As noted below, it is also linked to work-related factors such as commitment to the organization and job-related factors such as job satisfaction. Work centrality is also a variable of interest because it is one of the few work-related factors applicable to those not currently in the workforce; work centrality determines how individuals act both within and outside of the workplace (Alvesson et al., 2008).

While evidence remains wanting, existing research suggests these emerging factors show significant promise as influencers of employment behavior and preferences. Like age discrimination, these emerging variables can be classified as psychosocial because they capture psychological responses to the social organization of work.

#### Work Centrality

Work centrality is important to consider because it influences job attitudes and behaviors (Bal and Kooij, 2011). Work centrality refers to “individual beliefs regarding the degree of importance that work plays in their lives” (Walsh and Gordon, 2008, p. 46). Individuals with high work centrality identify more strongly with their work, and see work as more important in their life, than individuals with low work centrality. Research suggests that work centrality is positively associated with job satisfaction (Dutton et al., 2010) and work engagement (Schaufeli et al., 2008), and negatively associated with intention to retire (Harpaz, 2002). Additionally, work centrality appears to be especially important among older workers. Research by Armstrong-Stassen and Schlosser (2008) reveals that work centrality is positively related to older workers’ orientations toward, and propensity to, engage in development activities at work. Relatedly, older workers with high work centrality are more motivated to invest in their relationship with the organization (forming relational rather than transactional psychological contracts) and to remain with the company (Bal and Kooij, 2011). According to Bal and Kooij (2011, p. 518), “people with high work centrality are able to negotiate a relational contract with the organization, which consequently makes them more satisfied with their job, more engaged in their work, and less inclined to leave the organization”; and the relationship between work centrality and psychological contracts is “especially strong for older workers.”

#### Person-Job Fit

Person-job fit refers to the match between the abilities of a person and the demands of a job (Edwards, 1991). Research related to person-job fit indicates that the greater the congruence between the person and their job, the more positive the individual and organizational outcomes. Conversely, person-job misfit is associated with higher levels of depression (Caplan, 1987), lower job satisfaction and organizational commitment, and higher turnover intentions (O’Reilly et al., 1991) - each of which is associated with early retirement (Sejbaek et al., 2012). Moreover, other research suggests that individuals report greater intentions to leave their jobs (Maynard et al., 2006) and engage in job search behaviors when misfit exists (Feldman and Turnley, 1995). One study also suggests the relationship between person-job fit and job satisfaction may be stronger for older workers (Krumm et al., 2013). To date, research examining person-job fit has examined intention to leave and actual turnover, generally. The importance that person-job fit plays within the context of retirement decisions remains largely unknown.

#### Work-Life Conflict

Work-life conflict (WLC) refers to “a form of interrole conflict in which the role pressures from the work and family (and other non-work) domains are mutually incompatible in some respect” (Greenhaus and Beutell, 1985, p. 77). Longitudinal studies and meta-analyses show that WLC leads to lower levels of job satisfaction (Kossek and Ozeki, 1998; Shockley and Singla, 2011), low organizational commitment, and increased intentions to leave (Allen et al., 2000; Garcia et al., 2014). The meta-analysis of Allen et al. (2000) also showed that WLC may be responsible for poor health outcomes; a known influencer of early retirement (Australian Bureau of Statistics, 2017). However, fewer studies have examined WLC among older workers (Allen and Shockley, 2012) and its effects on their retirement decisions. One exception is a body of work based on the Wisconsin Longitudinal Study, where WLC was found to predict preferences to retire (Raymo and Sweeney, 2006) and early retirement (Kubicek et al., 2010). These authors found that the effects of WLC on early retirement were partly due to lower levels of job satisfaction (Kubicek et al., 2010), indicating a potential explanatory pathway. A cross-sectional study by Garcia et al. (2014) showed that increased WLC was associated with both preferences and intention to retire, illustrating that WLC may be an important determinant of employment participation and preferences among older workers.

### Established and Emerging Factors as a Function of Gender and Financial Pressure

The research examining whether established and emerging factors differentially affect retirement decisions for men and women, or those experiencing different levels of financial pressure, remains under-developed, despite increasing research and policy interest in employment participation. We examine gender because women tend to retire earlier than men (De Preter et al., 2013; Australian Bureau of Statistics, 2017), possibly because of the cumulative effect established and emerging factors have on their employment participation (Baird et al., 2012). We examine financial pressure because of its relationship with employment participation. Those experiencing more or less financial pressure are inclined to face quite different options in relation to employment participation/retirement (Warren, 2015). Developing a greater understanding of these issues will enable us to create more effective, targeted policies directed toward promoting employment participation among older workers, while taking account of their gender and financial pressure.

#### Gender

Women are working longer, but they are still retiring earlier than men (Austen and Ong, 2013; Warren, 2015). Literature suggests that caring responsibilities tend to push women into retirement and encourage men to remain employed (e.g., Meng, 2012; O’Loughlin et al., 2017), fulfilling their traditional “carer” and “breadwinner” roles, respectively (Loretto and Vickerstaff, 2013). If these traditional gender roles are still being enacted, certain “push” factors (see De Preter et al., 2013) should have a greater effect on women’s employment participation, relative to men’s, as they compound existing social pressures on women to retire earlier. In support of this argument, studies suggest that having a spouse that is already retired (Moen et al., 2001), age discrimination (Australian Human Rights Commission, 2015) and poor mental health (Paradise et al., 2012) push more women out of the workforce than men. Thus, it is hypothesized that other established and emerging factors may have a similar effect.

#### Financial Pressure

There has been considerable focus on the factors that force financially disadvantaged workers out of employment, including their low occupational status (Radl, 2013) and the negative health effects of relatively hazardous working conditions (Pit et al., 2010). It is plausible that those under financial pressure are more likely to perceive retirement as unaffordable (McManus et al., 2007). In fact, the most commonly reported reason for returning to the workforce after initial retirement is “financial need” (42%) (Australian Bureau of Statistics, 2017). Thus, factors such as poor health, caregiving and work conditions may have a limited effect on employment decisions for workers who are under financial pressure because they have little choice but to remain at work (Garcia et al., 2014). The financial burden associated with caregiving and poor health may even intensify their need to continue working. In contrast, workers under relatively less financial pressure may prefer to leave work if their health is poor or if their working conditions are not to their liking (Garcia et al., 2014). Older workers are likely to make different decisions as a result of different levels of financial pressure; thereby demanding more nuanced and targeted policy responses in order to prolong employment.

### Summary and Hypotheses

The literature reviewed suggests that both established and emerging factors will affect employment participation and preferences among older workers. Moreover, employment participation among older workers is likely to vary in accordance with their gender and financial pressures as predicted below.

#### Employment Status

Poor physical health, having an injury, caregiving, reports of age discrimination, poor mental health, and lower work centrality will:

H1a: Decrease the likelihood of being employed.

However, these relationships will be:

*H1b: Stronger for women*,
H1c: Stronger for those under less financial pressure.

#### Employment Preferences for Those Currently Employed

Poor physical health, having an injury, caregiving, reports of age discrimination, poor mental health, lower work centrality, person-job misfit and work-life conflict will:

H2a: Decrease the likelihood of preferring to remain employed.

However, these relationships will be:

*H2b: Stronger for women*,
H2c: Stronger for those under less financial pressure.

#### Employment Preferences for Those Currently Employed

Poor physical health, having an injury, caregiving, perceptions of insufficient job skills, reports of age discrimination, poor mental health, and lower work centrality will:

H3a: Decrease the likelihood of preferring to re-enter employment.

However, these relationships will be

*H3b: Stronger for women*,
H3c: Stronger for those under less financial pressure.

Post-GFC data is used to test the research hypotheses as it allows us to examine the relationships of primarily non-financial established and emerging factors with employment behaviors and preferences during a time when financial constraints would be expected to drive decision-making. Although Australia has arguably recovered from the GFC, it is facing increasing income inequality (Kaplan et al., 2018) and housing unaffordability (Daniel et al., 2018) that, if left unchecked, will create substantive financial pressure for those who cannot secure appropriate employment. As such, the findings will identify specific factors that may boost employment participation among older workers and facilitate the development of more effective policies for organizations and governments.

## Materials and Methods

### Participants

This study is drawn from a larger project examining the health and workforce participation of Australians aged between 45 and 65. The national survey data reported here were collected using computer-aided telephone interviewing (CATI) between February and June 2009. An ISO and industry-accredited social research company selected a random sample, stratified for gender and age group. Contact information was drawn from a database containing telephone numbers for approximately 4.8 million Australian households. A demographic analysis of the database conducted by an independent demographic consultancy indicated that it was representative of the socioeconomic and geographic distribution of the Australian population, other than extremely isolated communities. The overall sample of 1,541 participants was stratified by employment status and age (860 in paid employment, 681 not in paid employment). Approximately 20% of those not in paid employment indicated they were actively looking for employment.

### Measures

#### Dependent Variables

The three dependent variables were employment status (0 = not employed, 1 = employed), workers’ employment preference (0 = prefer to retire, 1 = prefer paid work) and non-workers’ employment preference (0 = prefer retirement, 1 = prefer paid work). In the survey, those in paid employment were asked if they would prefer to be retired (yes or no). Those not in paid work were asked to describe how important it was for them to have a paid job in the future (on a five-point scale from one to five). As the responses to this question were bi-modal, those scoring “1” or “2” were categorized as preferring retirement (coded 0) and the remainder were categorized as preferring paid employment (coded 1). These cut points were chosen to best balance the sample size for this dichotomous variable.

#### Independent Variables

The established variables included in this research are physical health, mental health, injury status, caregiving, age discrimination and perceived job skills. *Physical health* and *mental health* are assessed with composite scores derived from the SF-12 (Ware et al., 2002). Participants were also asked whether they have had an *injury* that limited their daily activities, and responses were coded “0” for injury and “1” for no injury. For *caregiving*, participants were asked whether they had a caring responsibility for a family member or friend, and responses were coded as either yes (coded 1) or no (coded 0). *Age discrimination* was assessed with the mean score of four items (Banas, 2007) on five-point scales (alpha = 0.81). These items assessed discrimination with respect to finding a job, being denied promotion, being denied a position of leadership or being in a lower-status position because of their age. Items were worded according to employment status. For example, those not currently working for pay responded to the statement, “Since my last job I have had difficulty finding employment because of my age,” whereas those currently in employment were presented with the statement, “I have difficulty finding paid work because of my age.” Those who were not employed were asked the extent to which they perceived *lack of skill* to be a barrier to re-entering employment. Responses were coded on a five-point scale with higher scores reflecting stronger perceptions of skill barriers to re-entering employment.

The emerging variables were work-life conflict, work centrality and person-job fit, which were all were assessed with five-point Likert scales ranging from strongly disagree (1) to strongly agree (5). *Work-life conflict* was measured with three items; (Kopelman et al., 1983: alpha = 0.65, e.g., “After work, I come home too tired to do some of the things I’d like to do”). *Work centrality* was assessed by responses to a single item: “I would enjoy having a paid job even if I did not need the money” (Svallfors et al., 2001). The acceptable use of single item measures to assess psychological constructs have been supported by Wanous et al. (1997) in their meta-analysis of single item measures for job satisfaction. For those currently in the workforce, self-reported *person-job fit* (Saks and Ashforth, 1997) was assessed with four items; for example, “My knowledge, skills, and abilities match the requirements of my job,” “My job is a good match for me” (alpha = 0.83).

The moderating variables were gender and financial pressure. *Gender* was measured with a dichotomous variable coded “0” for female and “1” for male. *Financial pressure* was assessed with the mean score of three items (e.g., “I have money left over at the end of the month” and “I make just enough money to make ends meet”) from the Pressure, Disorganization and Regulatory Failure scale (alpha = 0.65) (Bohle et al., 2015). A median split on the financial pressure composite was used as a grouping variable to reflect higher and lower financial pressure groups.

The control variables were financial dependents, marital status, age, and years out of employment (for those not currently working for pay). Participants were asked how many of the people in their household were wholly, or partly, financially dependent on them. This four-point variable ranged from 0 to 3 or more financial dependents. *Marital status* was measured with a dichotomous variable (not married = 0, married = 1) and *age* was measured continuously in years. Those not currently in employment were also asked how many years they had been out of paid employment.

### Analysis

Three binary logistic regressions (Mplus 7.1) were used to test the relationships between the independent variables and employment status, employment preferences for those currently in employment, and the employment preferences of those not in employment. These analyses were performed separately for men and women and for lower and higher financial pressure groups to test for moderation effects. Differences in cross-group unstandardized regression coefficients were tested with Mplus’ multiple-group analysis using the recommended known-class approach (Muthén and Muthén, 2012, p. 20) in which cross-group unstandardized regression coefficients are forced to be equal. If the equality constraint significantly worsens the model fit, the two coefficients are considered statistically different and a moderation effect is inferred (Muthén and Muthén, 2012). Variables were selected for cross-group testing if the 90% confidence intervals for the unstandardized regression coefficients did not overlap, which indicates a statistically significant interaction effect (Payton et al., 2003).

## Results

Descriptive statistics for the study variables are shown in Table 1.

**Table 1:** Descriptive statistics according to employment status.

Variables	Level for categorical variables	*N* (percentage)	Mean (SD)
**Dependent variables**			


Employment status	Employed	860 (55.8)	


	Not employed	681 (44.2)	
Preference for those in employment	Prefers to be employed	543 (63.1)	


	Prefers to not be employed	317 (36.9)	


Preference for those not in employment	Prefers to be employed	325 (47.7)	


	Prefers to not be employed	357 (52.3)	


**Independent variables**			


Physical health (0–100) Full sample			45.61 (11.88)


Employed			48.63 (9.38)


Not employed			41.81 (13.5)


Mental health (0–100) Full sample			49.52 (10.66)


Employed			50.89 (9.64)


Not employed			47.79 (11.60)


Injury Full sample	Yes	328 (21.3)	


	No	1213 (78.7)	


Employed	Yes	172 (20.0)	


	No	688 (80.0)	


Not employed	Yes	156 (22.9)	


	No	525 (77.1)	


Caregiving Full sample	Yes	510 (33.1)	


	No	1031 (66.9)	


Employed	Yes	244 (28.3)	


	No	616 (71.6)	


Not employed	Yes	266 (39.1)	


	No	415 (60.9)	


Age discrimination (1–5) Full sample			1.67 (1.02)


Employed			1.68 (1.05)


Not employed			1.65 (0.98)
Insufficient skills (1–5) Not employed			2.72 (1.60)
Work centrality (1–5) Full sample			3.29 (1.42)
Employed			3.41 (1.34)
Not employed			3.15 (1.50
Person-job fit (1–5) Employed			3.97 (0.92)
Work-life conflict (5) Employed			2.62 (1.09)
Financial pressure Full sample			2.91 (1.17)
Employed			2.74 (1.17)
Not employed			3.14 (1.13)
Gender Full sample	Male	640 (41.5)	
	Female	901 (58.5)	
Employed	Male	397 (46.2)	
	Female	463 (53.8)	
Not employed	Male	243 (35.7)	
	Female	438 (64.3)	
Age Full sample			55.51 (5.49)
Employed			54.68 (5.10)
Not employed			56.56 (5.79)
Financial dependents Full sample	No dependents	921 (59.8)	
	One dependent	318 (20.6)	
	Two or more dependents	302 (19.6)	
Employed	No dependents	438 (50.9)	
	One dependent	204 (23.7)	
	Two or more dependents	218 (25.3)	
Not employed	No dependents	483 (70.9)	
	One dependent	114 (16.7)	
	Two or more dependents	84 (12.3)	
Marital status Full sample	Married/de facto	1017 (66.0)	
	Not living with partner	524 (34.0)	
Employed	Married/de facto	598 (69.5)	
	Not living with partner	262 (30.5)	
Not employed	Married/de facto	419 (61.5)	
	Not living with partner	262 (38.5)	
Years since last worked Not employed			7.93 (8.68)


According to the bivariate correlations (Table 2), being in paid employment was associated with better physical health (*r* = 0.29, *p* < 0.01), mental health (*r* = 0.15, *p* < 0.01), fewer caregiving responsibilities (*r* = -0.11, *p* < 0.01), stronger work centrality (*r* = 0.09, *p* < 0.01) and less financial pressure (*r* = -0.17, *p* < 0.01). The preference to remain employed increased as physical health (*r* = 0.12, *p* < 0.01) and person-job fit improved (*r* = 0.16, *p* < 0.01). Higher levels of work centrality (*r* = 0.34, *p* < 0.01) and lower levels of work-life conflict (*r* = -0.20, *p* < 0.01) were also associated with the preference to remain employed. Employed caregivers also tended to prefer working (*r* = 0.08, *p* < 0.05). For those not currently employed, the preference to re-enter the workforce was associated with poorer mental health (*r* = -0.15, *p* < 0.01) and a positive evaluation of one’s skills (*r* = 0.11, *p* < 0.01). Those wanting to return to work also tended to be under greater financial pressure (*r* = 0.24, *p* < 0.01) compared to those who preferred retirement. They also reported higher levels of perceived age discrimination (*r* = 0.25, *p* < 0.01).

**Table 2:** Bivariate Pearson’s correlations between all variables.

		1	2	3	4	5	6	7^1^	8	9	10	11^2^	12^2^	13	14	15	16	17^1^
Employment status	1																	
Employment preference (employed)	2	.																
Employment preference (not employed)	3	.	.															
Physical health	4	0.29**	0.12**	-0.02														
Injury (no)	5	0.04	0.08*	0.00	0.34**													
Caregiving (yes)	6	-0.11**	0.08*	0.07	-0.02	-0.07*												
Perceived job skills^1^	7	.	.	0.11**	-0.04	0.05	-0.01											
Mental health	8	0.15**	0.07*	-0.15**	0.12**	0.09**	-0.09**	-0.13**										
Age discrimination	9	0.02	-0.04	0.25**	-0.08**	-0.01	0.03	0.12**	-0.17**									
Work centrality	10	0.09**	0.34**	0.30**	0.00	0.03	0.04	0.05	-0.03	0.03								
Person-job fit^2^	11	.	0.16**	.	0.12**	0.08*	0.00	.	0.12**	-0.33**	0.23**							
Work-life conflict^2^	12	.	-0.20**	.	-0.14**	-0.15**	0.09**	.	-0.31**	0.12**	-0.13**	-0.14**						
Financial pressure	13	-0.17**	-0.05	0.24**	-0.23**	-0.09**	0.04	0.13**	-0.24**	0.22**	0.00	-0.14**	0.17**					
Gender (male)	14	0.11**	-0.06	0.03	0.06*	-0.04	-0.14**	-0.09*	0.08**	0.07**	-0.02	-0.09*	0.05	-0.03				
Financial dependents	15	0.21**	0.05	0.18**	0.15**	0.01	0.10**	0.00	-0.03	-0.01	0.06*	-0.05	0.18**	0.04	0.14**			
Age	16	-0.17**	0.02	-0.41**	-0.01	0.01	-0.04	-0.13**	0.13**	-0.04	-0.07**	0.10**	-0.17**	-0.74**	0.04	-0.35**		
Years out of work^1^	17	.	.	-0.20**	-0.15**	0.00	-0.03	0.21**	-0.01	-0.15**	-0.09*	.	.	0.00	-0.14**	-0.11**	0.07	
Marital status (married)	18	0.08**	0.04	-0.10*	0.14**	0.05	-0.04	-0.03	0.15**	-0.11**	-0.01	0.09**	0.00	-0.15**	0.10**	0.22**	0.04	-0.07


*^∗∗^*p* < 0.01, ^∗^*p* < 0.05. 1 = not employed sample (*N* = 681), 2 = employed sample (*N* = 860).*

### Employment Status by Gender (Hypotheses 1a and 1b)

For both men and women, the likelihood of being employed increased as physical and mental health improved, and rates of caregiving decreased (see Table 3 for ORs). Contrary to expectations, higher perceived age discrimination increased the likelihood of being in paid employment for women, but there was no relationship for men. Having an injury increased the likelihood of men being out of the workforce, but not for women. Having financial dependents, a secure financial position, stronger work centrality and younger age increased the likelihood of being in paid employment for both sexes after controlling for other variables. However, multiple-group analysis showed no evidence of moderation according to gender.

**Table 3:** Logistic regression for the likelihood of being employed according to gender (*N* = 1,504).

	Women			Men		
		
	b	OR	ß	b	OR	ß
Physical health	0.04***	1.04	0.26	0.06***	1.06	0.32
Injury (yes)	-0.33	0.72	-0.06	-0.60*	0.55	-0.11
Caregiving (yes)	-0.50**	0.61	-0.12	-0.96***	0.39	-0.19
Age discrimination	0.25**	1.29	0.12	0.11	1.11	0.05
Mental health	0.02**	1.02	0.13	0.03**	1.03	0.12
Work centrality	0.16**	1.17	0.11	0.15*	1.16	0.09
Financial pressure	-0.25***	0.78	-0.14	-0.29**	0.75	-0.15
Financial dependents	0.41***	1.51	0.15	0.41**	1.50	0.15
Age	-0.04**	0.96	-0.11	-0.13***	0.88	-0.30
Marital status (married)	-0.26	0.77	-0.06	0.46*	1.59	0.09
Nagelkerke *R*^2^	20.30			34.10		


*b, unstandardized regression coefficient; OR, odds ratio; ß, standardized regression coefficient, ^∗^*p* < 0.05, ^∗∗^*p* < 0.01, ^∗∗∗^*p* < 0.001. Not in employment coded 0, in employment coded 1.*

### Employment Status by Financial Pressure (Hypotheses 1a and 1c)

Better physical and mental health, a lack of caregiving responsibility and having financial dependents were associated with higher rates of employment for both high and low financial pressure groups (see Table 4). Additionally, the likelihood of being employed decreased with age. Higher levels of work centrality and not having an injury increased the likelihood of employment for the lower financial pressure group. Multiple-group analysis provided no evidence for the moderating effects of financial pressure.

**Table 4:** Logistic regression for the likelihood of being employed according to financial pressure (*N* = 1,504).

	Higher			Lower		
	financial pressure			financial pressure		
		
	b	OR	ß	b	OR	ß
Physical health	0.04***	1.04	0.27	0.06***	1.06	0.31
Injury (yes)	-0.32	0.73	-0.07	-0.56*	0.57	-0.10
Caregiving (yes)	-0.58**	0.56	-0.14	-0.66***	0.52	-0.15
Age discrimination	0.18*	1.20	0.10	0.11	1.12	0.05
Mental health	0.04***	1.04	0.20	0.02*	1.02	0.08
Work centrality	0.04	1.04	0.03	0.23***	1.26	0.16
Gender (male)	0.05	1.05	0.01	0.31	1.36	0.07
Financial dependents	0.56***	1.75	0.22	0.24*	1.27	0.09
Age	-0.04*	0.96	-0.10	-0.09***	0.91	-0.24
Marital status (married)	-0.10	0.91	-0.02	0.06	1.07	0.01
Nagelkerke *R*^2^	21.40			24.70		


*b, unstandardized regression coefficient; OR, odds ratio; ß, standardized regression coefficient, ^∗^*p* < 0.05, ^∗∗^*p* < 0.01, ^∗∗∗^*p* < 0.001. Not in employment coded 0, in employment coded 1.*

### Employment Preferences for Those in Paid Employment: Differences by Gender (Hypotheses 2a and 2b)

Good physical health and caregiving responsibilities were associated with the preference to be employed for males (see Table 5). Having financial dependents was a significant correlate of the preference to remain in employment for women. Work centrality was positively associated with employment preferences for men and women but decreasing work-life conflict and improving person-job fit were only associated with women’s preferences to be employed. Moderation analysis showed that the relationship between caregiving and employment preferences was stronger for men (X^2^ difference = 4.97, df = 1, *p* < 0.027), while the relationship between person-job fit and employment preferences was stronger for women (X^2^ difference = 9.01, df = 1, *p* = 0.003).

**Table 5:** Logistic regression for employed participants: likelihood of preferring to remain in employment according to gender (*N* = 824).

	Women			Men		
		
	b	OR	ß	b	OR	ß
Physical health	0.01	1.01	0.06	0.03*	1.03	0.13
Injury (yes)	-0.02	0.98	0.00	0.31	1.36	0.06
Caregiving (yes)^#^	0.03	1.03	0.01	0.93**	2.54	0.19
Age discrimination	0.16	1.17	0.08	0.00	1.00	0.00
Mental health	0.00	1.00	-0.01	0.00	1.00	0.01
Work centrality	0.66***	1.94	0.40	0.41***	1.51	0.27
Work-life conflict	-0.51***	0.60	-0.25	-0.25*	0.78	-0.14
Personal-job fit^#^	0.49***	1.64	0.20	-0.09	0.91	-0.04
Financial pressure	-0.05	0.96	-0.03	0.01	1.01	0.00
Financial dependents	0.45*	1.56	0.16	-0.04	0.97	-0.02
Age	0.02	1.02	0.04	0.00	1.00	0.00
Marital status (married)	-0.13	0.88	-0.03	0.43	1.53	0.09
Nagelkerke *R*^2^	34.40			16.80		


*b, unstandardized regression coefficient; OR, odds ratio; ß, standardized regression coefficient, ^∗^*p* < 0.05, ^∗∗^*p* < 0.01, ^∗∗∗^*p* < 0.001. ^#^Denotes significant interaction effect. Prefer to not be employed coded 0, prefer to be employed coded 1.*

### Employment Preferences for Those in Paid Employment: Differences by Financial Pressure (Hypotheses 2a and 2c)

Physical health, caregiving responsibilities and work centrality were positively associated with preference to be employed for those under more financial pressure (see Table 6). For the lower-pressure group, workers with financial dependents, stronger work centrality, lower work-life conflict and higher person-job fit were independently associated with the preference to be employed. Moderation analysis suggested that work-life conflict was a stronger predictor of the preference to not remain employed for the lower-pressure group (X^2^ difference = 5.15, df = 1, *p* = 0.023).

**Table 6:** Logistic regression for employed participants: likelihood of preferring to remain in employment according to financial pressure (*N* = 824).

	Higher			Lower		
	financial pressure			financial pressure		
		
	b	OR	ß	b	OR	ß
Physical health	0.03*	1.03	0.17	0.02	1.02	0.06
Injury (yes)	0.00	1.00	0.00	0.12	1.13	0.02
Caregiving (yes)	0.76*	2.15	0.18	0.07	1.07	0.01
Age discrimination	0.04	1.04	0.02	0.09	1.09	0.04
Mental health	0.01	1.01	0.07	0.00	1.00	0.01
Work centrality	0.39***	1.47	0.26	0.62***	1.86	0.38
Work-life conflict^#^	-0.14	0.87	-0.08	-0.52***	0.60	-0.25
Personal-job fit	0.07	1.07	0.03	0.26*	1.30	0.11
Financial dependents	-0.06	0.94	-0.03	0.35*	1.43	0.13
Age	-0.04	0.96	-0.09	0.03	1.03	0.06
Gender (male)	-0.04	0.96	-0.01	-0.25	0.78	-0.06
Marital status (married)	-0.15	0.86	-0.04	0.20	1.22	0.04
Nagelkerke *R*^2^	16.00			31.10		


*b, unstandardized regression coefficient; OR, odds ratio; ß, standardized regression coefficient, ^∗^*p* < 0.05, ^∗∗^*p* < 0.01, ^∗∗∗^*p* < 0.001. ^#^Denotes significant interaction effect. Prefer to not be employed coded 0, prefer to be employed coded 1.*

### Employment Preferences for Those Not in Paid Employment: Differences by Gender (Hypotheses 3a and 3b)

Higher levels of perceived age discrimination in a previous job were associated with preferences to be re-employed among both genders (see Table 7). Higher levels of financial pressure, younger age, stronger work centrality and less time spent out of work were independently associated with men’s and women’s preferences to be re-employed. No interaction effects according to gender were identified.

**Table 7:** Logistic regression for currently not employed participants: likelihood of preferring to re-enter employment according to gender (*N* = 678).

	Women			Men		
		
	b	OR	ß	b	OR	ß
Physical health	0.00	1.00	-0.01	0.03	1.03	0.13
Injury (yes)	-0.02	0.98	0.00	0.23	1.26	0.04
Caregiving (yes)	0.22	1.25	0.05	-0.41	0.67	-0.07
Lack skills	0.10	1.11	0.07	0.10	1.11	0.06
Age discrimination	0.47**	1.60	0.16	0.44**	1.55	0.19
Mental health	0.01	1.01	0.05	0.00	1.00	-0.01
Work centrality	0.42***	1.52	0.27	0.34**	1.40	0.19
Financial pressure	0.26**	1.30	0.13	0.51**	1.67	0.22
Financial dependents	0.26**	1.30	0.07	0.31	1.36	0.09
Age	-0.13***	0.88	-0.33	-0.20***	0.82	-0.41
Marital status (married)	-0.22	0.81	-0.05	-0.42	0.66	-0.08
Years since last worked	-0.05***	0.95	-0.19	-0.05***	0.95	-0.13
Nagelkerke *R*^2^	41.00			50.00		


*b, unstandardized regression coefficient; OR, odds ratio; ß, standardized regression coefficient, ^∗^*p* < 0.05, ^∗∗^*p* < 0.01, ^∗∗∗^*p* < 0.001. Prefer to not be employed coded 0, prefer to be employed coded 1.*

### Employment Preferences for Those Not in Paid Employment: Differences by Financial Pressure (Hypotheses 3a and 3c)

Increasing levels of perceived age discrimination were associated with the preference to re-enter the workforce for both groups (see Table 8). Higher levels of work centrality and younger age increased the likelihood that both groups would prefer to be employed. For the higher financial pressure group only, increasing time spent out of employment was associated with the preference to not be employed. Moderation analysis showed that this negative relationship was stronger for the group under greater financial pressure (X^2^ difference = 4.27, df = 1, *p* = 0.039).

**Table 8:** Logistic regression for currently not employed participants: likelihood of preferring to re-enter employment, according to financial pressure (*N* = 678).

	Higher			Lower		
	financial pressure			financial pressure		
		
	b	OR	ß	b	OR	ß
Physical health	0.01	1.01	0.07	-0.01	1.00	-0.03
Injury (yes)	0.21	1.23	0.04	0.02	1.02	0.00
Caregiving (yes)	0.16	1.17	0.03	-0.08	0.93	-0.02
Lack skills	0.12	1.12	0.08	0.08	1.08	0.06
Age discrimination	0.61***	1.84	0.26	0.33	1.39	0.11
Mental health	0.01	1.01	0.05	-0.01	0.99	-0.03
Work centrality	0.36***	1.43	0.22	0.43***	1.54	0.28
Gender (male)	0.26	1.30	0.05	-0.09	0.91	-0.02
Financial dependents	0.23	1.26	0.07	0.39	1.48	0.12
Age	-0.14***	0.87	-0.34	-0.15***	0.86	-0.36
Marital status (married)	-0.21	0.81	-0.04	-0.47	0.62	-0.10
Years since last worked^#^	-0.07^∗∗∗^	0.93	0.26	-0.02	0.98	-0.07
Nagelkerke *R*^2^	45.40			36.50		


*b, unstandardized regression coefficient; OR, odds ratio; ß, standardized regression coefficient, ^∗^*p* < 0.05, ^∗∗^*p* < 0.01, ^∗∗∗^*p* < 0.001. ^#^Denotes significant interaction effect. Prefer to not be employed coded 0, prefer to be employed coded 1.*

## Discussion

Partial support for the research hypotheses suggests that only selected established and emerging factors may play an important role in promoting employment participation among older workers. With respect to Hypotheses 1a and 2a, physical health, caregiving (established factors) and all three emerging factors were consistently associated with employment status and preferences, but injury and mental health status were not. Some of these relationships also varied according to gender and financial pressure. For women, there was a stronger relationship between person-job fit and the preference to remain in employment (Hypothesis 2b), whereas for men, caregiving increased their preference to remain employed. Work-life conflict was associated more with the preference to not be employed for workers who are under less financial pressure (Hypothesis 2c). Although there was no support for Hypotheses 3, the preference to re-enter employment decreased with time spent out of work for the high financial pressure group.

### Established Factors

In terms of the established determinants of employment participation and preferences, good physical health was among the strongest correlates of workforce participation, regardless of gender or financial pressure, as shown in other studies (e.g., Pit et al., 2010; Topa et al., 2018). While this finding is consistent with literature suggesting that poor health forces people into retirement (Dwyer and Mitchell, 1999), our additional analyses revealed the importance of gender and financial pressure in this relationship. For example, workers experiencing greater financial pressure, and men, were more likely to prefer to remain in employment if they were in better health, but there was no relationship among wealthier participants or women. Likewise, physical health had no bearing on participants’ preferences to re-enter the workforce irrespective of financial pressure or gender.

The other significant factor among the established determinants was caregiving. In line with previous research (Fine, 2012; Australian Human Rights Commission, 2013), caregiving was among the strongest correlates of non-employment for men and women, across both financial pressure groups. However, the relationship between caregiving and workers’ preferences to remain employed was stronger for men. This is consistent with prior research demonstrating that men are less likely to reduce hours or exit the workforce due to caregiving (Kröger and Yeandle, 2013), whereas women experience greater social pressure to give up work to provide care (Fine, 2012).

Although correlations indicated that the likelihood of being employed increased as mental health improved, consistent with extant research (e.g., Laaksonen et al., 2012), there was no association between mental health and retirement preferences in the regression analysis. It is likely that the relationship between mental health and employment preferences was explained by higher rates of caregiving, greater financial pressure, work-life conflict, lower person-job fit and perceived skill deficiency found in those with poorer mental health. Contrary to the findings of Paradise et al. (2012), there was no evidence to suggest that mental health affects employment participation and preferences in different ways for men and women.

Interestingly, injury was associated with employment status, but not preferences for employment. Having an injury increased the likelihood of men being out of the workforce, but not of women. This finding conflicts with earlier research (Crook et al., 1998), which indicated that return to work after an injury is slower for women compared to men. A possible explanation may relate to the strong physical demands associated with male-dominated occupations, such as construction, mining and manufacturing. Additionally, physically demanding work is likely to be more significant for mature-aged workers because physical work capacity (impacted by changes in cardiovascular and musculoskeletal capacity) declines with increasing age, and after 50 years the deterioration is more marked (Ilmarinen, 2001). In contrast, not having an injury increased the likelihood of employment among those experiencing less financial pressure. This finding is consistent with pre-existing literature, and echoes evidence indicating that people with greater financial resources consistently have better health outcomes than those with fewer resources (Smith, 2004).

The results suggest that although a lack of skill may be perceived as a barrier to re-employment, it has no influence on preferences to be re-employed. At face value, this appears inconsistent with previous research (e.g., Berg et al., 2017). However, these studies examined job skills and training as avenues for keeping people in work, rather than pulling people out of retirement. Perceived skill deficits were positively associated with reports of age discrimination, poor mental health and financial pressure, suggesting it may function as an indicator of broader disadvantage. Therefore, job skill development programs that are designed to keep workers in employment could target these groups.

Age discrimination showed weak but significant correlations with employment status and preferences to re-enter employment, but in the opposite direction to that expected based on pre-existing findings (e.g., von Hippel et al., 2011). For females, and those in financially adverse circumstances, age discrimination marginally increased the likelihood of being in employment. Higher levels of discrimination were associated with the preference to re-enter the workforce for both genders and in the higher financial pressure group. These findings may reflect the heightened salience of age discrimination for those seeking re-employment, whereas age discrimination is less salient to those who are not actively seeking re-employment. It is also important to note that, for those not currently employed, the measure may not have captured aspects of discrimination that led to premature retirement, particularly given the silent manner in which discrimination operates (Australian Human Rights Commission, 2015).

### Emerging Factors

Work centrality was associated with employment participation and preferences; a finding that is consistent with other studies demonstrating that high work centrality is associated with lower turnover (Bothma and Roodt, 2012) and intention to retire (Harpaz, 2002). Some studies suggest that work centrality declines as people approach retirement age (Misumi and Yamori, 1991). However, in our relatively narrow sample of 45- to 65-year olds, there were only minor differences according to either age, years out of employment or employment status. Pending further research, this finding suggests that work centrality could function as a widely relevant intervention point for those in the second half of their working life.

Person-job fit was associated with the preference to remain in employment. While prior literature indicates that person-job fit impacts job satisfaction, organizational commitment (O’Reilly et al., 1991) and intention to leave one’s job (Maynard et al., 2006), it may also precipitate early retirement. However, after controlling for the other established and emerging factors, the relationship between person-job fit and preference to remain in employment was only significant for women and those under less financial pressure. This finding could reflect pressure on women to retire early (Loretto and Vickerstaff, 2013); in order to continue working, a woman’s job has to fit with her non-work commitments.

Consistent with the literature (Kubicek et al., 2010; Garcia et al., 2014), WLC was associated with the preference to leave employment for all groups except those under financial pressure. For this group, retirement may be more likely to be perceived as unaffordable. Therefore, factors such as reduced work-life conflict may be less salient drivers for staying employed than, for instance, a caregiving commitment (see Table 6). In line with the meta-analysis of Allen et al. (2000), WLC was also associated with poorer health outcomes as well as lower work centrality and person-job fit, representing potential intervention points, as discussed below.

### Implications

These findings are central to facilitating the development of more targeted initiatives to promote older workers’ employment participation. In terms of developing organizational initiatives, our findings highlight the importance of leveraging the established and emerging factors identified in this study. More specifically, workplace policies need to address physical health and injury, caregiving, age discrimination, work centrality, work-life conflict and person-job fit.

Organizational policies related to occupational health and safety (OHS) may be especially important in promoting physical health and avoiding injuries among workers, along with employee assistance programs (EAP) related to maintaining health through exercise and diet (Grunseit et al., 2017). Our findings suggest that such policies and programs should be targeted, in particular, toward men and workers under greater financial pressure because improved physical health among these groups is likely to contribute to their continued employment participation.

In Australia, the legal right to request flexible work arrangements has been extended to specifically include older workers (aged 55+ years) and those with carer responsibilities for older people or those with a disability/illness (Cooper and Baird, 2015). However, there is no guarantee that a request will be granted or that it will be implemented consistently across workplaces. Our findings suggest that organization-specific caregiver policies could contribute to older men and women participating more strongly in employment, and enable women and those under greater financial pressure to continue participating in employment for longer periods of time. Such caregiver policies should encompass flexible working-time arrangements and assistance/advice regarding provision of care for dependents and/or onsite care options (Baird et al., 2012; Cooper and Baird, 2015). To facilitate and prolong employment participation among older workers, age discrimination needs further and systematic consideration at both the policy and organizational level to address what appear to be persistent ageist attitudes and behaviors in workplaces (Australian Human Rights Commission, 2015; O’Loughlin et al., 2017).

The provision of flexible work arrangements is also one of a number of strategies for reducing work-life conflict and promoting employee wellbeing more generally (e.g., Anderson et al., 2002; Zheng et al., 2016). Specific examples include the provision of increased schedule control (Kelly et al., 2011), supervisor support (Anderson et al., 2002) and organizational support in balancing work and family commitments (Anderson et al., 2002; Kossek et al., 2011). However, as Malbon and Carey (2017) suggest, such arrangements can be detrimental to health if they inadvertently create longer working hours, reduce separation between home and work life, or if they lead to insecure (“flexible”) working conditions. These facets of flexible work arrangements should therefore be considered in the design of new interventions.

Organizations can strengthen work centrality through the enhancement of job resources, such as social support at work, career-building opportunities, participation in decision-making and performance feedback (De Braine and Roodt, 2011). Other research suggests that work design and job enrichment processes can be used to enhance work centrality, particularly via increased job autonomy, interest, variety and responsibility (Sharabi and Harpaz, 2010). Our findings suggest that such initiatives will be useful in promoting employment participation among all older workers, regardless of gender or financial pressure.

Additionally, organizations can improve person-job fit by paying greater attention to “fit” during recruitment and selection activities (e.g., through enhanced anticipatory socialization processes) (Anderson and Ostroff, 1997), and focusing on redesigning jobs to improve job-holder fit and/or engaging in training to improve the skills, knowledge and abilities of the job-holder to ensure better fit (Bauer et al., 1998; Maynard et al., 2006; Kalleberg, 2008; Truxillo et al., 2012). Increasing fit may produce particular benefits for women and those experiencing less financial pressure, thereby increasing their employment participation.

For wealthier workers and women in particular, the main drivers of employment preferences were WLC, work centrality and person-job fit, even during a period of financial pressure created by the GFC. This pattern of results supports previous calls to focus on improving the psychosocial work environment as a potential government initiative to prolong employment, rather than increasing the pension age (Taylor et al., 2014; Davies et al., 2017; Noone and Bohle, 2017). Indeed, policies in other countries, including Great Britain (Health Safety Executive, 2000) and Canada (Canadian Standards Association, 2013), incorporate the psychosocial work environment into strategies to promote worker health, albeit without a specific focus on older workers. Such initiatives would complement emerging government-backed Australian programs to promote workers’ mental health, including Beyond Blue, 2016 and Safe Work Australia’s older workers program (Butterworth et al., 2017). However, it is becoming increasingly clear that collaboration will be required to achieve this goal (Australian Government, 2014), particularly through the provision of an evidence base and policy to assist employers in creating healthy workplaces.

Although Topa et al.’s (2018) model was used more for variable selection than theory testing, our findings do provide some useful insights for their model. With greater statistical control in place, aspects of the work environment (person-job ft) family pull (caregiving, work-life conflict) and individual factors (health, work centrality) were all independently associated with employment preferences depending on gender and financial pressure. This means that no dimension can be singled out as most important. Although further research is needed, one implication is that interventions could be particularly effective if they are holistic, treating individual circumstances, family life and working environment as inter-related rather than discrete components.

Findings also suggest the importance of a multi-disciplinary or multi-level approach to improving employment participation. The emerging factors, which cover psychological responses to the work environment, were equally as important as socioeconomic (i.e., financial pressure) and sociodemographic factors (e.g., caregiving) as potential influencers of employment status and preferences. This suggests that future research should better explore the psychological mechanisms involved in retirement decision making. For example, future time perspective, self-efficacy and decision-making style may act as potential mediators in the antecedent – employment decision relationship. To date, psychological factors like these have been largely studied in relation to retirement planning (Earl et al., 2015; Rafalski and Andrade, 2016) rather than the specific decision to work or retire. Indeed, Topa et al.’s (2018) meta-analysis of antecedents for early retirement did not examine any psychological factors excepting mental health. This is important because further study into psychological mechanisms could reveal new opportunities for intervention and policy change.

The findings of this research offer insight into the variables associated with older Australians’ employment participation and preferences, with potential implications for improved management practices and policy development. However, the study has notable limitations that could be addressed in future research. In terms of methodology, the cross-sectional design limits inferences of causality and raises the possibility that common method variance influenced the results (Podsakoff et al., 2003). These limitations could be avoided by using longitudinal and mixed methods designs. Further, although work centrality emerged as an important factor associated with participation and preferences, additional information may be needed to implement direct management or policy intervention. For instance, interventions to increase work centrality may be enhanced by developing better understanding of its correlates and antecedents, such as gender and parental identities, parental responsibilities, and job satisfaction (Mannheim et al., 1997; Gaunt and Scott, 2017), and identifying those most likely to respond to intervention. Other limitations that should be acknowledged are the use of dichotomous variables (Cohen, 1983) and the inability to determine the importance of particular established and emerging factors across different industries. This is an important topic for future research along with cross-national research to test the generalizability of the findings and sociological enquiry into generational change in gendered push factors.

In sum, this research makes an important contribution as it identifies a set of established and emerging factors that may promote workforce participation for older workers, depending on their gender and financial circumstances. Subsequently, these findings will facilitate the development of organization and government policies that more precisely target older workers, including their gender and financial circumstances, in order to prolong older workers’ employment participation. Pending further research, these findings reflect critical focal points that may prolong employment and encourage working longer; thereby creating more viable employment options that will reduce rates of involuntary retirement.

## Ethics Statement

This study was carried out in accordance with the recommendations of the National Statement on Ethical Conduct in Human Research (2007), the University of Sydney Human Research Ethics Committee with verbal informed consent from all subjects via telephone interview. All subjects gave verbal informed consent in accordance with the Declaration of Helsinki. The protocol was approved by the University of Sydney Human Research Ethics Committee.

## Author Contributions

JN conceptualized the manuscript, performed the statistical analysis, and contributed to writing the literature review, method, results, and discussion sections. AK assisted in the conceptualization of the manuscript and contributed to writing the literature review and discussion sections. KO assisted in the conceptualization of the manuscript and contributed to writing the literature review and discussion sections. MMN assisted in the conceptualization of the manuscript, managed the data collection, and contributed to writing the method and introduction sections. PB contributed to conceptualization and development of the manuscript, led the research project, and was a chief investigator on the related grant. MM contributed to writing the introduction and discussion sections.

## Conflict of Interest Statement

The authors declare that the research was conducted in the absence of any commercial or financial relationships that could be construed as a potential conflict of interest.
